# Protocol for the biochemical isolation of endosomal membranes associated with endoplasmic reticulum in HeLa cells

**DOI:** 10.1016/j.xpro.2026.104563

**Published:** 2026-05-11

**Authors:** Juliane Da Graça, Etienne Morel

**Affiliations:** 1 Université Paris Cité, INSERM UMR-S1151, CNRS UMR-S8253, Institut Necker Enfants Malades, 75015 Paris, France; 2 Interfaculty Institute of Bioengineering and Global Health Institute, École Polytechnique Fédérale de Lausanne, 1015 Lausanne, Switzerland

**Keywords:** Cell Biology, Cell Membrane, Cell separation/fractionation

## Abstract

Endosome-endoplasmic reticulum contact sites (EERCS) are highly dynamic membrane interfaces that regulate trafficking and signaling, yet biochemical approaches to isolate them remain nonexistent, unlike established protocols for other types of contacts. Here, we present a protocol to isolate and purify endosomal membranes associated with endoplasmic reticulum membranes from cultured mammalian cells using subcellular fractionation and a two-step density-gradient centrifugation. We describe steps for cell expansion, post-nuclear supernatant collection, and the isolation of light membranes and EERCS.

For complete details on the use and execution of this protocol, please refer to Da Graça et al.^1^

## Highlights

• Procedures for post-nuclear supernatant preparation from HeLa cells
• Workflow for two-step density-gradient fractionation of endosomal membranes
• Instructions for isolating ER-endosome contact-site membranes
• Guidance on preserving native ER-endosome contact sites during isolation

## Abstract (editor highlights)

Publisher’s note: Undertaking any experimental protocol requires adherence to local institutional guidelines for laboratory safety and ethics.

## Graphical abstract

**Figure:**
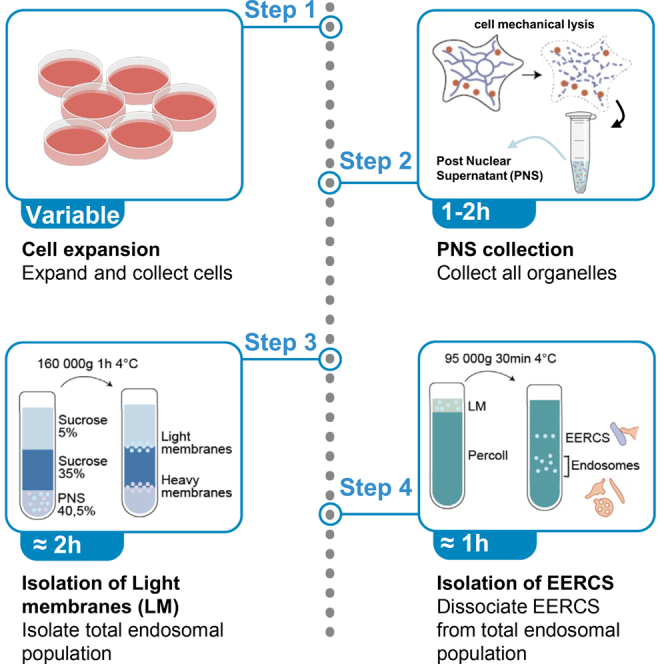

## Before you begin

This protocol describes the isolation and purification of total endosomal membranes associated with endoplasmic reticulum (ER) membranes, called EERCS for endosome-ER contact sites, from mammalian cells using subcellular fractionation and density-gradient centrifugations. These steps are then followed by validation of EERCS enrichment using biochemical approach.

Membrane contact sites between organelles are dynamic and transient structures^2^^,^^3^^,^^4^ that are difficult to isolate biochemically. While protocols for isolating ER-mitochondria contact sites have been established for many years,^5^ comparable approaches for other contact sites, including ER-endosome interfaces, remain limited. The present protocol describes the specific steps used to isolate EERCS fractions from HeLa cells, developed in Da Graça et al.^1^ However, this workflow can also be applied to other mammalian cell lines (e.g., HEK293 or MEFs) after optimization of homogenization conditions and provided that you start from a large quantity of material.

Using this protocol, you will isolate an ER–endosome contact site (EERCS)–enriched fraction containing both ER-resident tethering proteins (e.g., VAPs, VMP1, KTN1) and endosomal markers (e.g., RAB GTPases, EEA1). This fraction can be directly compared, in a volume-to-volume manner, with a companion fraction enriched exclusively in endosomal proteins that are not engaged in ER contact. Together, these two fractions allow quantitative assessment of the proportion of endosomes physically associated with the ER under a given condition. In our previous study,^1^ this fractionation strategy enabled the quantification of dynamic changes in ER–endosome contacts across distinct endosomal subpopulations during cellular stress responses. The protocol is therefore well suited for interrogating how membrane contact sites reorganize in response to physiological or experimental perturbations.

To preserve organelle integrity and maintain native membrane contact sites throughout the procedure, the entire workflow must be performed on ice or at 4°C. Before beginning, ensure that all buffers, reagents, tubes, rotors, and centrifugation equipment are fully pre-cooled. Maintaining low temperature at every step is essential to prevent membrane remodeling, vesicle fusion, or loss of contact site–associated proteins.

### Innovation

This protocol provides the first biochemical strategy to selectively isolate endosomal membranes engaged in contact with the ER from the total endosomal pool. By enriching for ER-associated endosomes while preserving native contact sites, it enables direct comparison of their molecular composition and dynamics. This approach offers a powerful complement to imaging-based methods and expands the experimental toolkit for studying ER–endosome communication.

### Cell culture

**Timing: Variable**

It is recommended to culture a large number of cells (from 4x10^7^ to 6 x 10^7^ in total) before initiating the isolation steps. Adequate starting material is essential for obtaining reproducible yields of both the ER–endosome contact site–enriched fraction and the corresponding endosome-only fraction.

1. HeLa cells are cultured in MEM, supplemented with GlutaMAX (41090093; Thermo Fisher Scientific), 10% FCS, and non-essential amino acid mix (11140035; Gibco, Thermo Fisher Scientific) and incubated at 37°C with 5% CO_2_.
2. Grow cells in 150-mm dishes, ensuring that they remain as a uniform monolayer throughout culture. For each condition, prepare at least 4 × 10^7^ cells to obtain sufficient material for a robust purification yield. In general, the larger the starting cell mass is, the more efficient and reproducible the isolation of ER–endosome contact site–enriched fractions will be.

### Material preparation

3. Prepare 3mM imidazole pH7.4, HB-EGTA buffer, 35% sucrose and 62% sucrose solutions, Percoll basal medium and 30% Percoll solution as described in the materials and equipment section and keep at 4°C.

***Note:*** All solutions can be prepared in advance except for the 30% Percoll which needs to be fresh.

**CRITICAL:** Ensure to have fresh Percoll stock solution.

**CRITICAL:** The use of EGTA is essential to eliminate residual Ca^2+^ from all buffers. Fluctuations in cytosolic Ca^2+^ levels promote the dissociation of ER–endosome contact sites; therefore, maintaining Ca^2+^-free conditions throughout the procedure is critical to preserve native ER–endosome interactions.

4. Pre-cool all centrifuges at 4°C.
5. Pre-cool ultracentrifuge rotor, holders and tubes at 4°C.
6. Prepare a large bucket of ice and place all collection tubes (15-mL and 1.5-mL), rubber adapters, and syringes on ice to ensure they remain fully chilled throughout the procedure.

## Key resources table

**Table:** 

REAGENT or RESOURCE	SOURCE	IDENTIFIER
**Antibodies**		

Anti- Calnexin	BD Bioscience	Cat#: 610523
Anti- VAP-A	ProteinTech	Cat#: 15275-1-AP
Anti- VMP1	Cell Signaling	Cat#: 12929
Anti- EEA1	BD TransLab	Cat#: 610456
Anti- Rab5	Cell signaling	Cat#: 2143
Anti- Rab7	Abcam	Cat#: ab50533
Anti- Rab11	Cell Signaling	Cat#: 2413 S
Centrifuges, rotors and tubes	–	–
Optima MAX-XP Benchtop Ultracentrifuge	Beckman Coulter	Cat#: 393315
TLS-55 Swinging-Bucket Rotor	Beckman Coulter	Cat#: 346936
1 mL Open-Top Thickwall Polycarbonate Tube	Beckman Coulter	Cat#: 343778
Eppendorf 5810 R Centrifuge	Eppendorf	Cat# 5811 000.518

**Experimental models: Cell lines**		

HeLa cells	ATCC	CCL2
Reagents		
Percoll	Sigma	Cat# P4937
Sucrose	Sigma	Cat# S9378
Immidazol	Sigma	Cat# 1202

**Other**		

22G Needle	BD (Becton Dickinson)	Cat# 305155
1 mL Syringe	BD (Becton Dickinson)	Cat# 309628
DABCO pen	Electron Microscopy Sciences (EMS)	Cat# 17998-10
Pasteur Pipette	VWR	Cat# 14672-380
Cell Scraper	Sigma	Cat# 41122103

## Materials and equipment

**Table:** HB-EGTA Solution

Reagent	Final concentration	Amount
Sucrose	8%	17.1 g
100mM EGTA	0.1mM	15 μL
3mM Immidazole pH7.4	3mM	q.s. 200mL
**Total**	**N/A**	**200 mL**

**Table:** 35% Sucrose

Reagent	Final concentration	Amount
Sucrose	35%	40.3 g
3mM Immidazole pH7.4	3mM	q.s. 100mL
**Total**	**N/A**	**100 mL**

**Table:** 62% Sucrose

Reagent	Final concentration	Amount
Sucrose	62%	40.25 g
3mM Immidazole pH7.4	3mM	q.s. 50mL
**Total**	**N/A**	**50 mL**

**Table:** Percoll basal medium

Reagent	Final concentration
Mannitol	225mM
HEPES pH7.4	25mM
EGTA	1mM

**Table:** Percoll 30% Solution

Reagent	Final concentration	Amount
Percoll	30%	2.4 mL
Percoll basal medium	**N/A**	5.6 mL
**Total**	**N/A**	**8 mL**

## Step-by-step method details

### HeLa cell collection and preparation

**Timing: ≈ 30–60 min**

1. Harvest cells.
  a. Place culture dish on ice.
  b. Remove culture media and wash twice with 5 mL cold PBS.
  c. In 150mm dish, add 6mL of PBS and gently scrap cells in a circular motion with cold cell scraper to detach cells without lysate them.
    ***Note:*** Push cells forward, don’t run over them. Keep similar direction and don’t pass multiple times on the same spot. The whole process should not exceed 2min per dish.
  d. Collect cell fragments using wide plastic Pasteur pipette in cold 15mL tubes.
2. Prepare cells for lysis.
  a. Centrifuge the cells at 125 × g for 5 min at 4°C (≈800 rpm in an Eppendorf 5810 R).
  b. Carefully remove the supernatant and add 3 mL of HB-EGTA buffer. Gently resuspend the pellet using a wide-bore pipette.
    ***Note:*** Avoid narrow pipette tips, which can shear or lyse cells. Do not exceed 2 gentle up-and-down motions to preserve organelle integrity.
  c. (Optional) Pool cells from identical experimental conditions into the same 15-mL tube to increase the final purification yield.
  d. Centrifuge at 750 × g for 10 min at 4°C (≈2,000 rpm in an Eppendorf 5810 R).

### Preparation of post-nuclear supernatant

**Timing: ≈ 30–60 min**

3. Lyse cells.
  a. Remove the supernatant and add an equal volume of HB EGTA buffer relative to the volume of the cell pellet.
    ***Note:*** Aim for a 1:1 ratio (50% pellet + 50% HB EGTA). Use at least 200 μL of HB EGTA to ensure proper homogenization.
  b. Homogenize the suspension by performing 1–5 gentle aspiration–dispense cycles with a 1,000 μL wide-bore pipette tip, avoiding bubble formation.
  c. Further homogenize the whole mixture using a pre chilled 22G needle, applying up to 3 slow, controlled strokes while avoiding bubbles.
    **CRITICAL:** Apply moderate, steady force; expelling the sample should take approximately 30–60 s. Excessive shear may disrupt organelles and compromise the purification, whereas insufficient force may require additional strokes and reduce homogenization efficiency.
  d. Prepare a microscopy slide by drawing circles with a DABCO pen and placing 80 μL drops of HB EGTA inside each circle.
  e. Add 5 μL of the homogenized mixture to the HB EGTA drops and assess homogenization using inverted tissue-culture microscope.
    ***Note:*** Nuclei should appear round and free of attached organelles or plasma membrane, as shown in Figure 1A. Organelles should move freely with the liquid flow. If nuclei appear swollen or misshaped, homogenization was too strong and the preparation must be restarted from Step 1. After homogenization, the mixture can be kept on ice for several hours if necessary.
    **Figure 1:**
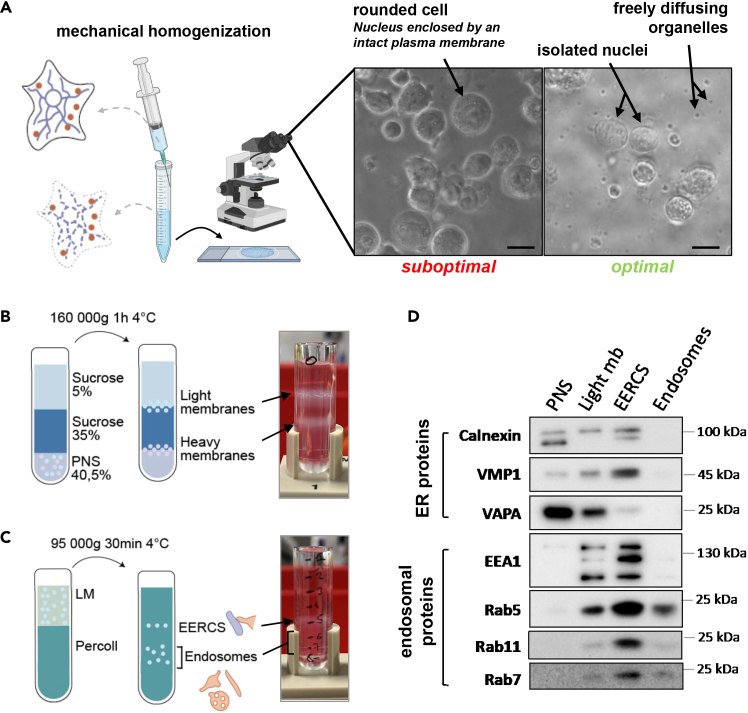
Biochemical purification of endosome-ER contact sites (EERCS) (A) Schematic overview of the mechanical homogenization procedure. Cells are lysed using a 22G needle, and lysis quality is assessed by bright-field microscopy. The “suboptimal” example shows intact, round cells in which the nucleus remains surrounded by plasma membrane, indicating insufficient homogenization. The “optimal” example shows naked nuclei with free-floating organelles in the surrounding medium, indicating successful lysis. Scale bars: 10 μm. (B) Schematic and representative image of the first ultracentrifugation step (150,000 × g, 1 h, 4°C). The post-nuclear supernatant (PNS), adjusted to 40.6% sucrose, is layered beneath a 35% sucrose cushion and a 5% sucrose top layer. After centrifugation, light membranes—corresponding to the total endosomal population—are recovered at the 5%–35% sucrose interface, while heavy membranes sediment at the 35%–40.6% interface. (C) Schematic and representative image of the second ultracentrifugation step (95,000 × g, 30 min, 4°C). Light membranes diluted in 3 mM imidazole are layered on top of a 30% Percoll solution. After centrifugation, a distinct band corresponding to the EERCS fraction is recovered at the interface, while the raw endosomal population sediments below. (D) Representative western blot of fractions collected throughout the purification workflow: post-nuclear supernatant (PNS), light membranes (Light mb), EERCS fraction, and raw endosomal fraction (Endosomes). Membranes were probed with antibodies against ER proteins (Calnexin, VMP1, VAP-A) and endosomal proteins (EEA1, Rab5, Rab11, Rab7), confirming the co-enrichment of ER tethering proteins and endosomal markers in the EERCS fraction relative to the raw endosomal fraction.
  f. Centrifuge the homogenate at 750 × g for 10 min at 4°C (≈2,000 rpm in an Eppendorf 5810 R).
4. Collect PNS.
  ***Note:*** After centrifugation, two phases should be visible: the light white slightly opaque upper layer corresponds to PNS, and the viscus opaque pellet are nuclei.
  a. Collect PNS, here PNS#1, ideally using a cut 1000μL pipette tip.
  b. Resuspend carefully the pellet with 80μL of HB-EDTA.
    ***Note:*** Do not exceed more than 10 times of up and down pipetting.
  c. Centrifuge cells at 1180 g, 10min, 4°C (2 500rpm with centrifuge Eppendorf 5810 R).
  d. Collect upper phase, here PNS#2, ideally using a cut 1000μL pipette tip.
  e. Pool PNS#1 and PNS#2.
  f. Equilibrate the PNS at 40.6% of sucrose by adding an equal volume of 62% sucrose. Keep aliquot of PNS of western blot detection.
    Mix the solution using a cut 1,000-μL pipette tip, slowly pipetting up and down until the sucrose is fully dissolved; this step may require several minutes. Do not vortex or shake the tube. Sucrose concentration can be verified using a refractometer: acceptable values range from 39% to 42%, with 40.6% providing the most optimal gradient performance.

### Isolation of light membranes

**Timing: ≈ 2 h**

5. Ultracentrifugation #1.
  a. In precooled TLS-55 tubes, pour <500μL of 40.6% PNS (generally 300μL), then using a 26G needle, pour very gently 500μL of 35% sucrose first and 500μL of HB-EGTA second, as shown in Figure 1B.
    **CRITICAL:** Three distinct phases need to visible. The quality of the gradient will determine the efficiency of enrichment in endosomal populations.
  b. Centrifuge samples at 150 000 g using a TLS-55 rotor, 1 h, 4°C (50 000rpm with Optima MAX-XP Benchtop Ultracentrifuge).
    ***Note:*** After centrifugation, tubes should look like in Figure 1B. Collect light membranes as soon as possible after the end of the ultracentrifugation, otherwise floating lipids will sink and contaminate the endosomal population.
  c. Slowly remove the fat-containing surface of the gradient using a cut tip.
  d. Using a cut 200 μL pipette tip, gently aspirate 150 μL of the first white interface while performing slow spiral movements just above the fraction. Transfer the collected material to a separate tube. This fraction corresponds to light membranes (i.e., the total endosomal population).
  e. (Optional) The second fraction corresponding to heavy membranes (Mitochondria, Golgi, plasma membrane) can be collect using the same method after the removal of the intermediate sucrose to avoid any contamination.

### Isolation of EERCS

**Timing: ≈ 1 h**

6. Ultracentrifugation #2.
  a. Dilute 150 μL of light membranes with 450 μL of 3 mM imidazole (3:1 imidazole:LM ratio).
    ***Note:*** Mix using a cut 1,000-μL pipette tip, slowly pipetting up and down until the solution is fully homogenized. Avoid vortexing or shaking the tube.
  b. In precooled TLS-55 tubes, pour 1mL of 30% Percoll, then 450 μL of diluted light membranes.
    **CRITICAL:** Layer the diluted light-membrane fraction extremely carefully. This step is highly sensitive: the light membranes must remain fully on top of the 30% Percoll layer, forming a sharp, clean interphase. If the layers mix or the interphase becomes diffuse, the isolation of EERCS will fail.
  c. Centrifuge samples at 95 000 g using a TLS-55 rotor, 30min, 4°C (38 000rpm with Optima MAX-XP Benchtop Ultracentrifuge).
    ***Note:*** After centrifugation, the tubes should appear as shown in Figure 1C, with a distinct band corresponding to EERCS. Mark the position of this band on the tube, then carefully remove a small volume from the top of the gradient to facilitate precise fraction collection.
  d. Collect 300μL the first white interface by doing slow spiral movements slightly on top of the fraction using a cut 1000μL, this fraction corresponds to EERCS.
  e. Remove 100μL of liquid to avoid any contamination between EERCS and the raw endosomal population.
  f. Collect the next 300 μL; this fraction corresponds to the raw endosomal population.
  g. Note: This fraction is not visible by eye and should be collected strictly by volume. The 300 μL volume was empirically defined based on enrichment of endosomal markers (e.g., Rab11). Collecting larger volumes may dilute the endosomal fraction and affect downstream analyses. Validation using endosomal markers is recommended when adapting the protocol. Dilute each fraction to a final concentration of 1× Laemmli buffer containing 25 mM DTT. Heat the samples for 5 min at 95°C and proceed with western blot analysis.

## Expected outcomes

This protocol yields two biochemically distinct fractions: an EERCS-enriched fraction and a remaining endosomal fraction. The EERCS fraction should display strong enrichment for ER–endosome contact site proteins (e.g., VMP1, VAP-A, KTN1) together with endosomal membrane markers such as Rab5, Rab11, EEA1, and Rab7. In contrast, the endosomal fraction retains primarily endosomal proteins with minimal levels of ER-tethering factors (see Figure 1D).

Western blot analysis of equal volumes of both fractions enables calculation of an EERCS/endosome ratio for each protein of interest. This ratio reflects the proportion of a given endosomal population that is engaged in ER contact at the time of collection, providing a quantitative readout of ER–endosome communication dynamics.

As an example, after 15 min of starvation, the EERCS/endosome ratio of Rab5 increases markedly compared with the baseline level observed in control conditions, reflecting the acute recruitment of early endosomes to ER contact sites. After 60 min of starvation, the Rab5 ratio returns to baseline, whereas the Rab11 ratio increases, indicating a temporal shift toward recycling-endosome engagement (Figures 1K and 1L in Da Graça et al;^1^). These dynamics can be quantified across multiple independent experiments to assess how specific endosomal populations are regulated during cellular stress or other biological conditions.

Starting from two 150-mm dishes of confluent HeLa cells per condition, expect to obtain a low-yield but contact-site-enriched EERCS band after Percoll centrifugation. The yield is inherently limited by the transient nature of membrane contact sites and should not be compared with classical organelle-purification protocols. Increasing the amount of starting material will improve the final yield.

## Limitations

Several limitations should be considered when applying this protocol.

First, EERCS are highly dynamic and transient, and their biochemical isolation captures only a snapshot of the contact-site landscape at the moment of cell lysis. Any delay or fluctuation in temperature during the procedure can alter the composition of the recovered fractions, potentially leading to underestimation or loss of contact-site–associated proteins.

Second, the protocol was optimized for HeLa cells and may require substantial adaptation for other cell types, particularly primary cells, non-adherent cells, or cells with reduced endosomal content. Parameters that may require re-optimization include homogenization strength, sucrose-gradient composition, and the amount of starting material.

Third, the EERCS fraction obtained here represents a mixed population of endosomal membranes associated with the ER, without distinguishing between ER subdomains (e.g., ER exit sites, smooth ER) or between endosomal subtypes (e.g., Rab5^+^ early endosomes vs. Rab11^+^ recycling endosomes). Further discrimination requires marker-specific immunoblotting or complementary approaches such as proximity labeling (e.g., split-TurboID, Da Graça et al.^1^) or imaging-based quantification of contact sites.

Fourth, this protocol does not provide spatial information on EERCS organization within cells. For a comprehensive characterization of ER–endosome contact dynamics, it should be combined with microscopy-based methods such as proximity ligation assays, expansion microscopy, or live-cell imaging.

Finally, the low yield of the EERCS fraction limits its suitability for mass-spectrometry–based proteomics unless additional enrichment steps or substantial upscaling of starting material are implemented.

## Troubleshooting

### Problem 1

Nuclei appear swollen or misshaped after homogenization.

### Potential cause

Excessive force was applied during homogenization with the 22G needle, leading to organelle disruption.

### Potential solution

Discard the preparation and restart from Step 1. Apply more moderate force, reduce the number of strokes, and monitor lysis under the microscope after each stroke to avoid over-homogenization.

### Problem 2

Most cells remain intact after homogenization.

### Potential cause

Homogenization force was insufficient.

### Potential solution

Perform 1–2 additional strokes with the 22G needle and re-evaluate under the microscope. Continue until nuclei appear round and free of attached organelles or plasma membrane.

### Problem 3

Three distinct phases are not visible in the sucrose gradient after loading.

### Potential cause

The gradient layers mixed during pipetting.

### Potential solution

Restart gradient preparation. Layer the 35% sucrose and HB-EGTA solutions even more slowly using the 26G needle. Keep tubes perfectly still and horizontal during loading, and avoid any vibration of the bench or centrifuge area.

### Problem 4

No visible white interface at the light-membrane position after the first ultracentrifugation.

### Potential cause

Insufficient starting material or poor gradient quality.

### Potential solution

Use at least two confluent 150-mm dishes per condition. Verify sucrose-gradient quality before centrifugation. If needed, pool material from additional dishes.

### Problem 5

Fat layer sinks and contaminates the light-membrane fraction before collection.

### Potential cause

Delay between the end of ultracentrifugation and fraction collection.

### Potential solution

Collect the light-membrane fraction immediately after the centrifuge stops. Do not allow tubes to stand, as floating lipids rapidly sink and contaminate the endosomal population.

### Problem 6

No clean interphase between diluted light membranes and the Percoll solution, or diluted light membranes sink into Percoll.

### Potential cause

The diluted light membranes were loaded too quickly or with excessive force, disrupting the interphase.

### Potential solution

Load the diluted light membranes drop-by-drop against the tube wall, with extreme care. Ensure the Percoll solution is fully pre-cooled. A sharp, stable interphase is essential for successful EERCS isolation. Confirm that the 3:1 imidazole:light-membrane dilution was correctly prepared and fully homogenized before loading.

### Problem 7

No visible EERCS band after Percoll centrifugation.

### Potential cause

Insufficient starting material, loss of contact sites during the procedure, or incorrect Percoll concentration.

### Potential solution

Increase starting material by pooling multiple dishes. Ensure that all steps are performed strictly at 4°C and that delays between steps are minimized. Prepare a fresh 30% Percoll solution and verify its concentration.

### Problem 8

High cross-contamination between EERCS and raw endosomal fractions.

### Potential cause

Fractions were collected in volumes larger than specified, or the spacer volume between fractions was not removed.

### Potential solution

Collect exactly 300 μL per fraction. Ensure the 100 μL spacer volume between the EERCS and endosomal fractions is removed before collecting the endosomal fraction.

### Problem 9

Poor enrichment of ER-tethering proteins (e.g., VMP1, VAP-A) in the EERCS fraction.

### Potential cause

Membrane contact sites were disrupted by mechanical stress or temperature fluctuations.

### Potential solution

Avoid vortexing, agitation, or changes in ionic or redox conditions throughout the protocol. Maintain strict 4°C conditions and minimize total processing time.

### Problem 10

Protein degradation in the final fractions.

### Potential cause

Protease activity during fractionation.

### Potential solution

Add a protease inhibitor cocktail to all buffers before starting. Keep samples strictly on ice and minimize delays between steps.

## Resource availability

### Lead contact

Requests for further information and resources should be directed to and will be fulfilled by the lead contact, Etienne Morel (etienne.morel@inserm.fr).

### Technical contact

Technical questions on executing this protocol should be directed to and will be answered by the technical contact, Juliane Da Graca (juliane.dagraca@epfl.ch).

### Materials availability

This study did not generate new or unique reagents.

### Data and code availability

• This paper does not report original code.
• Any additional information required to reanalyze the data reported in this paper is available from the lead contact upon request.

## Acknowledgments

The authors warmly thank Professor Jean Gruenberg (University of Geneva, CH) for his advice and long-standing expertise. The endosomal fraction purification method employed in this study originates from pioneering work carried out in his laboratory. The authors also thank their colleagues at the MEMBRAMICS lab.

This work was supported by 10.13039/501100001677INSERM (Institut National pour la Santé et la Recherche Médicale), 10.13039/501100004794CNRS (10.13039/501100004794Centre National pour la Recherche Scientifique), Université Paris Cité, the 10.13039/501100001665French National Research Agency (ANR-17-CE140030-02, ANR-17-CE13-0015-003, ANR-22-CE14-0019, ANR-18-CE14-0006, and ANR-23-CE14-0041-01), and the French Foundation for Medical Research (10.13039/501100002915FRM, “labellisation équipe”). J.D.G. was the recipient of a doctoral fellowship from the French Ministry of Research/Université Paris Cité and a fourth-year PhD scholarship from the 10.13039/501100002915FRM.

## Author contributions

J.D.G. designed, conceptualized, and conducted the experiments. J.D.G. drafted the manuscript. E.M. supervised the work and revised the manuscript.

## Declaration of interests

The authors declare no competing interests
